# Amphotericin B-conjugated polypeptide hydrogels as a novel innovative strategy for fungal infections

**DOI:** 10.1098/rsos.171814

**Published:** 2018-03-14

**Authors:** Chang Shu, Tengfei Li, Wen Yang, Duo Li, Shunli Ji, Li Ding

**Affiliations:** 1 Key Laboratory of Drug Quality Control and Pharmacovigilance (China Pharmaceutical University), Ministry of Education, Nanjing 210009, People's Republic of China; 2 Department of Pharmaceutical Analysis, School of Pharmacy, China Pharmaceutical University, 24 Tongjiaxiang, Nanjing, 210009, People's Republic of China

**Keywords:** amphotericin B, polypeptide hydrogel, antifungal activity, biocompatibility

## Abstract

The present work is focused on the design and development of novel amphotericin B (AmB)-conjugated biocompatible and biodegradable polypeptide hydrogels to improve the antifungal activity. Using three kinds of promoting self-assembly groups (2-naphthalene acetic acid (Nap), naproxen (Npx) and dexamethasone (Dex)) and polypeptide sequence (Phe-Phe-Asp-Lys-Tyr, FFDKY), we successfully synthesized the Nap-FFDK(AmB)Y gels, Npx-FFDK(AmB)Y gels and Dex-FFDK(AmB)Y gels. The AmB-conjugated hydrogelators are highly soluble in different aqueous solutions. The cryo-transmission electron microscopy and scanning electron microscopy micrographs of hydrogels afford nanofibres with a width of 20–50 nm. Powder X-ray diffraction analyses demonstrate that the crystalline structures of the AmB and Dex are changed into amorphous structures after the formation of hydrogels. Circular dichroism spectra of the solution of blank carriers and the corresponding drug deliveries further help elucidate the molecular arrangement in gel phase, indicating the existence of turn features. The *in vitro* drug releases suggest that the AmB-conjugated hydrogels are suitable as drug-controlled release vehicles for hydrophobic drugs. The antifungal effect of AmB-conjugated hydrogels significantly exhibits the antifungal activity against *Candida albicans*. The results of the present study indicated that the AmB-conjugated hydrogels are suitable carriers for poorly water soluble drugs and for enhancement of therapeutic efficacy of antifungal drugs.

## 1. Introduction

Invasive fungal infection has a significant influence on animal and plant life, which is a major cause of mortality that kills approximately 1.5 million people every year [1]. Amphotericin B (AmB) is a broad-spectrum antifungal macrolide polyene antibiotic [2]. Its antifungal effect is exerted by forming complexes with membrane sterols to allow the increase in the permeability of the fungal membrane [3]. AmB is the most common antifungal agent used in clinical practice despite strong side-effects with low solubility and low bioavailability (less than 0.9%) [4]. Many researchers attempt to develop novel AmB formulations combined with nanoparticles, nanotubes, polymers etc. Current research works have indicated that poly-aggregated AmB formulations could reduce the toxicity and enhance the efficacy [5–7].

Peptide hydrogels, a hydrogel type resulting from self-assembly of amino acids in water solution, have emerged as the new class of attractive soft nanomaterials owing to their unique physiochemical characteristics [8]. Because the hydrogelators associate with each other only through non-covalent interactions, hydrogels are inherently biocompatible and biodegradable. So, they are attractive candidates for drug self-delivery [9]. Compared with biodegradable polymers, self-delivery hydrogels based on hydrogelators could change some shortcomings such as adjusting the pore size or networks of polymers, limited loading of drug molecules and functionalizing the polymers with drug molecules [10,11]. Hence, new strategies are needed to conjugate AmB with hydrogels in order to increase its efficacy, reduce its toxicity and to minimize the chance of fungal resistance to AmB.

**Figure 1.**
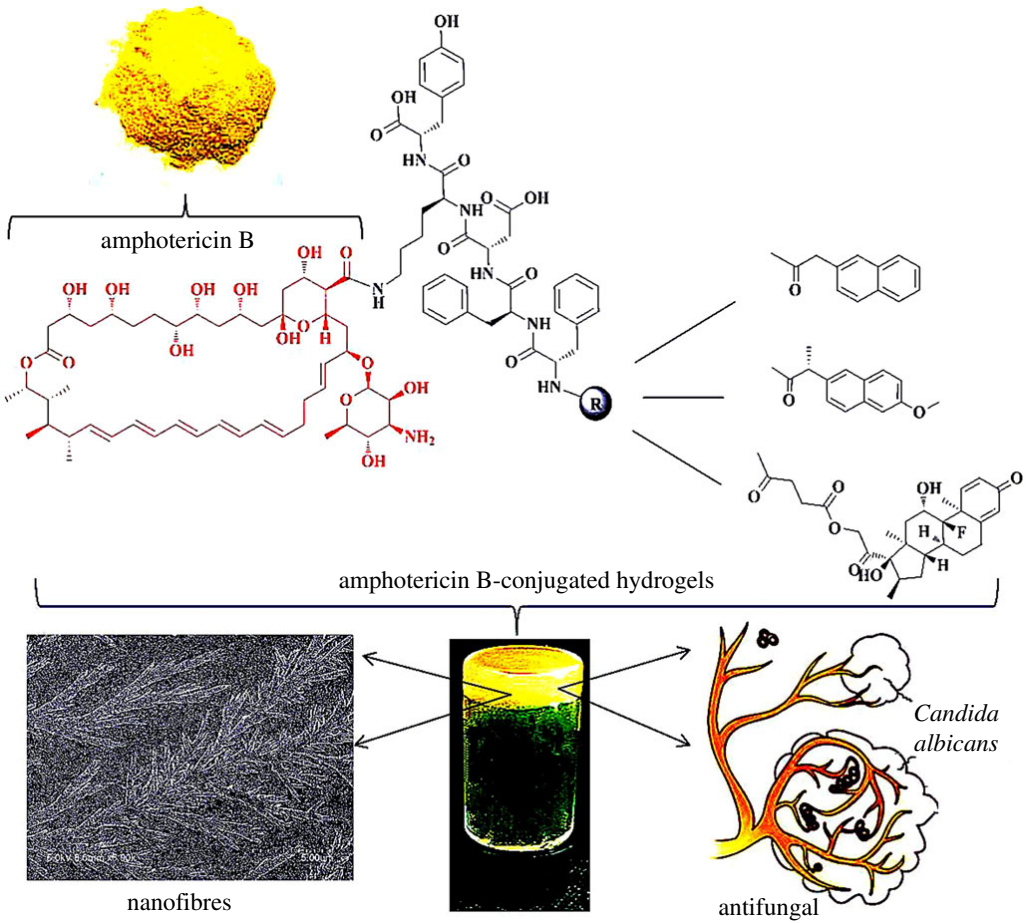
The illustration for antifungal properties of Nap-AmB, Npx-AmB and Dex-AmB hydrogels.

Considering the problems above, we design and synthesize several AmB-conjugates as molecular hydrogelators. The principles of the design are described as follows: (i) the peptide sequence of hydrogels contains Asp-Lys (DK) dipeptide fragments because it is crucial for antibacterial activity [12]—we hope that it can assist AmB to enhance antifungal activity; (ii) dipeptide of phenylalanine (FF) has been widely used to construct molecular hydrogelators; (iii) 2-naphthalene acetic acid (Nap) and naproxen (Npx) are frequently used to promote self-assembly—in the previous literature, Npx-FF fragments exhibit anti-inflammatory effect [13]; and (iv) dexamethasone (Dex) is an anti-inflammatory which is usually given alone or in combination with other antifungal drugs. Dex could reduce allergic reactions and the cell damage caused by AmB [14]. These are owing to the immunosuppressive effects and the varying permeability of cell membranes of Dex [15]. From the above, the Nap-FFDK(AmB)Y, Npx-FFDK(AmB)Y and Dex-FFDK(AmB) Y hydrogelators (figure 1) are synthesized (hereinafter to be referred as Nap-AmB, Npx-AmB and Dex-AmB hydrogels). Thus, chemical, physical and antifungal properties of AmB-conjugated hydrogels are examined.

## 2. Experiment

### 2.1. Materials

N-Fmoc-protected amino acids were obtained from GL Biochem Corporation Ltd (Shanghai, China). Nap, Npx, AmB, Dex, 1-hydroxybenzotriazole (HOBt), N,N-diisopropylethylamine (DIPEA), O-(Benzotriazol-1-yl)-N,N,N′, N′-tetramethyluronium tetrafluoroborate (TBTU), 4-dimethylamino-pyridine (DMAP) were obtained from Aladdin Reagent Corporation (Shang Hai, China). The purity of the AmB was 80% (750 µg mg^−1^). The purity of the Dex was 98%. The Trifluoroacetic acid (TFA) was purchased from Energy Chemical Corporation (Shanghai, China). The succinic anhydride was obtained from Sinopharm Reagent Co., Ltd (Shanghai, China). The chromatographic grade methanol and acetonitrile were purchased from Merck kGaA (Darmstadt, Germany). *Candida albicans* was a gift from the Department of Microbiology of China Pharmaceutical University. Peptone, glucose and agar were purchased from Beijing Laboratory Biology Technology Co., Ltd (Beijing, China).

### 2.2. Characterizations

Electrospray-ionization mass spectra were recorded using a Triple Quad 6410B instrument (America). Samples, which were presented as 1 mg ml^−1^ in dimethyl sulfoxide (DMSO), were diluted 100-fold (10 µl sample and 990 µl methanol). The proton spectra nuclear magnetic resonance (^1^H-NMR) experiments were performed on DMSO-d6 solution of samples at room temperature. The proton spectra were recorded on a Bruker (300 MHz, Germany). Cryo-transmission electron microscopy (TEM) images were taken using a Hitachi transmission electron microscope (Japan). Experiments were carried out using a field emission cryo-electron microscope operating at 80 kV. Thereafter, 5 µl of sample solution at 1.0 wt% concentration was applied on the grid. Grids with vitrified sample solutions were maintained in a liquid nitrogen atmosphere and then cryo-transferred into the microscope. Scanning electron microscopy (SEM) was used to characterize the microstructure of lyophilized hydrogel powder. SEM analysis was performed using SEM-4800 (Japan). Samples were attached to sample stubs and then viewed using an accelerating voltage at the magnification. The images were taken under inert condition with the electron microscope. The crystal structure of the product powder was analysed by a D8 advance powder X-ray diffractometer (PXRD, Bruker, Germany) where Cu radiation was used as an X-ray source. For the analysis, samples were placed in the glass sample holders and scanned from 3° to 40° at 40 kV operating voltage. The diffraction spectra were recorded. Circular dichroism (CD) spectra were recorded using a J-810 Chirascan spectropolarimeter (JASCO, Japan). The sample (0.1–1 mg ml^−1^ in water) was placed in a coverslip cuvette (0.1 mm thick). Spectra are presented with absorbance A at any measured point with a 1 nm step, 1 nm bandwidth, and 1 s collection time per step at 25°C.

### 2.3. Synthesis

The Nap-FFDKY, Npx-FFDKY and Fmoc-FFDKY hydrogelators were prepared by the solid-phase peptide synthesis (SPPS) using 2-chlorotrityl chloride resin (1.0–1.2 mmol g^−1^) and N-Fmoc-protected amino acids [16]. The resin swelled in dry dichloromethane (DCM) for 20 min, and was washed with dry DCM three times (every time for 2 min). Then the first amino acid (2 equiv.) was loaded onto resin with DIPEA for 2 h in a DCM solution. After being washed with DCM three times (every time for 2 min), the blocking solution (80 : 15 : 5 of DCM/MeOH/DIPEA) was added twice (every time for 10 min) to deactivate the unreacted sites. After being washed with dry N,N-dimethylformamide (DMF) five times (every time for 2 min), the resins were treated with 20% piperidine (in DMF) for 30 min to remove the protecting group. The next Fmoc-protected amino acid (2 equiv.) was coupled using HBTU (2 equiv.) and HOBt (2 equiv.) as the coupling reagent. These two steps were repeated to elongate the peptide chain, which was carried out by the standard SPPS protocol. In the final step, the peptide was cleaved with TFA for 2 h. Ice-cold diethyl ether was added, and then was centrifuged for 10 min at 4000 rpm. The resulting precipitates were dissolved in DMSO and purified by reverse-phase high preparative performance liquid chromatography (HPPLC, Agilent1200, America). The pure white Nap-FFDKY compound was obtained with a yield of 99.8%. ESI-MS: C_49_H_54_N_6_O_10_, calc.MW = 886.4, obsvd. [M + H]^+^ = 887.4. ^1^H-NMR (300 MHz, DMSO-d6). The pure white Npx-FFDKY compound was obtained with a yield of 99.3%. ESI-MS: C_51_H_58_N_6_O_11_, calc.MW = 930.4, obsvd. [M + H]^+^ = 931.2. ^1^H-NMR (300 MHz, DMSO-d6). The pure white Fmoc-FFDKY compound was obtained with a yield of 99.7%. ESI-MS: C_52_H_56_N_6_O_11_, calc.MW = 941.0, obsvd. [M]^+^ = 941.3. ^1^H-NMR (300 MHz, DMSO-d6).

After the AmB powder was dissolved in the DMF solution, 1.5 equiv. of Nap-FFDKY or Npx-FFDKY compound was added with 2.5 equiv. DIPEA. The resulting reaction mixture was stirred overnight protecting against exposure to light. At last, the product was purified by reverse-phase HPPLC. The pure yellow Nap-AmB hydrogelator compound was obtained with a yield of 58.7%. ESI-MS: C_97_H_129_N_7_O_25_, calc.MW = 1791.9, obsvd. [M + Na]^+^ = 1814.2. ^1^H-NMR (300 MHz, DMSO-d6). The pure yellow Npx-AmB hydrogelator compound was obtained with a yield of 55.7%. ESI-MS: C_99_H_133_N_7_O_26_, calc.MW = 1835.9, obsvd. [M + Na]^+^ = 1857.4. ^1^H-NMR (300 MHz, DMSO-d6).

The Dex (196.1 mg, 0.5 mmol) and succinic anhydride (117.1 g, 1.5 mmol) were dissolved in 10 ml of pyridine, then the DMAP (183.3 mg, 1.5 mmol) was added. After being stirred overnight at room temperature, the mixture was evaporated under reduced pressure. Ten millilitres of water was added. The mixture was stirred for 10 min and then centrifuged. The resulting precipitate was washed again with H_2_O three times. The white powder (Dex-SA) was obtained with a yield of 75.3%. After the AmB powder was dissolved in DMF solution, 1.5 equiv. of Fmoc-FFDKY compound was added with 2.5 equiv. DIPEA. The reaction mixture was stirred overnight protecting against exposure to light. The product was purified by reverse-phase HPPLC. The product was treated with 20% piperidine (in DMF) for 30 min to remove the protecting group. 1.5 equiv. of Dex-SA was added with 2.5 equiv. DIPEA. The mixture was stirred overnight avoiding light. At last, the result product was purified by reverse-phase HPPLC. The pure yellow powder Dex-AmB hydrogelator was obtained with a yield of 35.1%. ESI-MS: C_111_H_152_FN_7_O_31_, calc.MW = 2098.1, obsvd. [M + Na]^+^ = 2121.4. ^1^H-NMR (300 MHz, DMSO-d6).

### 2.4. Hydrogel formation

General procedure for the formation of hydrogels: all the compounds dissolved in phosphate-buffered saline (PBS, pH 7.4). 2 equiv. of Na_2_CO_3_ (0.1 mol l^−1^) were used to neutralize the carboxyl groups (─COOH) of compounds and adjust the pH to neutral. Then the hydrogels were formed at room temperature (22–25°C) or 37°C within 30 min.

### 2.5 Drug release

The 2.0 wt%, 3.0 wt% and 4.0 wt% of Nap-AmB, Npx-AmB and Dex-AmB hydrogels within the 0.3 ml PBS solution containing trypsin (1 µg ml^−1^) were prepared for drug release study. A quantity of 0.2 ml of the upper buffer solution was taken out and used to run HPLC at every 30 min and 0.2 ml of fresh PBS buffer solution containing trypsin (1 µg ml^−1^) was added back each time. The experiments were conducted at 37°C. The areas of peaks in HPLC spectra were used to determine the percentage of AmB or Dex released from their corresponding hydrogels. The liquid chromatography system (Agilent 1200 series, America) comprised Degasser G1322A, Quaternary pump G1312B, Thermostated auto sampler HiP-ALS G1367C, column oven G1316A and ultraviolet detector G1314A. Separation was achieved using a SepaxGP-C18 reversed-phase column (250 mm × 4.6 mm internal diameter, 5 µm particle size) (America). The mobile phase of AmB analysis was acetonitrile-water (43 : 57, containing 0.05% TFA) at a flow-rate of 1 ml min^−1^. Chromatography was carried out at 30°C and the detector wavelengths were set at 383 nm. The mobile phase of Dex analysis was acetonitrile-water (45 : 55, containing 0.05% TFA) at a flow rate of 1 ml min^−1^. Chromatography was carried out at 30°C and the detector wavelengths were set at 240 nm. The experiment was conducted in three parallel experiments. The release profiles of AmB and Dex from hydrogels is well described by the following exponential heuristic equation [17]:

2.1 $$M_{t}/M_{\infty }=k∙t^{n}.$$

*M_t_*/*M*_∞_ is the fractional drug release, *M_t_* is the amount of drug released at time *t*, *M*_∞_ is the maximum amount of drug released at time ∞, *t* is the release time, *k* is a rate constant of kinetic release and *n* is the diffusion exponent, characteristic of the drug release mechanism. For *n* < 0.5, it indicated that the drug release follows the Fickian diffusion, whereas the non-Fickian drug release process had a value of *n* between 0.5 and 1.

### 2.6. Antifungal activity

The minimum inhibitory concentration (MIC) of the Dex, AmB, Nap-FFDKY, Npx-FFDKY, Fmoc-FFDKY, Nap-AmB, Npx-AmB and Dex-AmB hydrogels was determined by a serial 2-fold dilution of test samples. Fourteen different concentrations of drugs and hydrogels were prepared in DMF solution. One millilitre of the fungal strain 10^18^ colony-forming unit (CFU)/tube was inoculated into each tube containing different concentrations (1 ml) of test samples and incubated at 28°C (200 rpm) for 24 h. The MIC was defined as the minimum concentration of the sample inhibiting the visible growth of the microorganism. The experiment was repeated at least three times. Based on the values of MIC, 0.1 ml of the fungal strain containing different concentrations of test samples which inhibited the visible growth of the microorganism was subcultivated on the Sabouraud's dextrose agar plates, and incubated at 28°C for 48 h. The minimum bactericidal concentration (MBC) determination was defined as the minimum concentration of the sample sterilizing the visible growth of the microorganism (lower than 5 CFU/plate). The experiment was repeated at least three times. Based on the values of MIC, the fungal strain containing 1 × MIC and 2 × MIC concentrations of test samples was incubated at 28°C. A test suspension containing 0.1 ml was taken out and used to evaluate fungus colonies at 0, 1, 2, 4, 6, 8, 12 and 24 h.

## 3. Results and discussion

### 3.1. Appearance images

**Figure 2.**
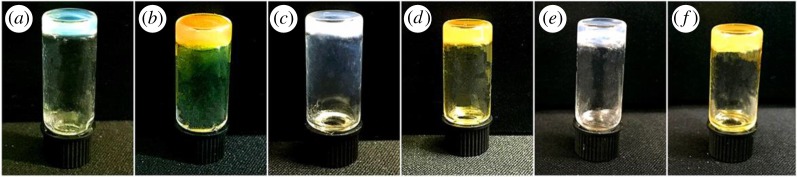
Appearance images of the (*a*) Nap-FFDKY, (*b*) Nap-AmB, (*c*) Npx-FFDKY, (*d*) Npx-AmB, (*e*) Fmoc-FFDKY and (*f*) Dex-AmB hydrogels (2.0 wt%).

Nap-AmB, Npx-AmB and Dex-AmB hydrogelators are highly soluble in different aqueous solutions, such as water, PBS solutions (pH = 7.4) and culture solutions. Figure 2 shows images of hydrogels in PBS solutions (2.0 wt%). The Nap-FFDKY, Npx-FFDKY and Fmoc-FFDKY hydrogels present almost white translucent gels (figure 2*a*,*c*,*e*). However, Nap-AmB, Npx-AmB and Dex-AmB hydrogels present yellow translucent gels (figure 2*b*,*d*,*f*). Gelation times are 3 min, 10 min and approximately 30 min for Nap-AmB, Npx-AmB and Dex-AmB hydrogels, respectively, at room temperature. The minimum gel concentration of Nap-AmB, Npx-AmB and Dex-AmB hydrogels needed for gelation is about 0.5, 1.0 and 2.0 wt%, respectively, in PBS solutions at 37°C.

### 3.2. Transmission electron microscopy and scanning electron microscopy micrographs

**Figure 3.**
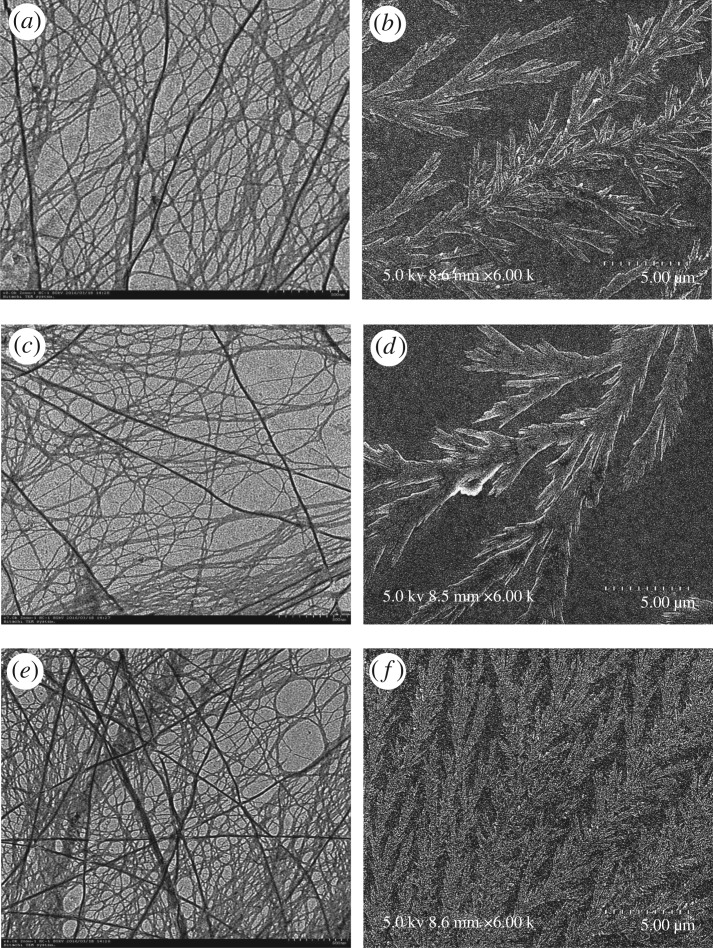
TEM micrographs of (*a*) Nap-AmB, (*c*) Npx-AmB, (*e*) Dex-AmB hydrogels (scale bar, 500 nm), and SEM micrographs of (*b*) Nap-AmB, (*d*) Npx-AmB, (*f*) Dex-AmB hydrogels (scale bar, 5 µm).

As shown in TEM micrographs, Nap-AmB hydrogels (figure 3*a*) afford large, straight and rigid nanofibres with a width of 20–50 nm. Npx-AmB hydrogels (figure 3*c*) exhibit long, narrow, and flexible nanofibres with a width of 10–30 nm, which tend to form bundles. The Dex-AmB hydrogels (figure 3*e*) show smaller diameter nanofibres that entangle to form a network with higher density than Nap-AmB and Npx-AmB hydrogels. The morphologies of SEM micrographs of Nap-AmB, Npx-AmB and Dex-AmB hydrogels are similar to those of TEM micrographs. It is noted that Nap-AmB (figure 3*b*) and Npx-AmB (figure 3*d*) hydrogels randomly assemble in dendritic clusters, while Dex-AmB hydrogels are observed as aquatic plants or feather structures (figure 3*f*). These morphological differences of the hydrogels indicate that the different head group (Nap-, Npx- and Dex-) at the N terminals of hydrogelators probably plays a role in their self-assembly. The self-assembly of Nap-AmB, Npx-AmB and Dex-AmB hydrogels would minimize AmBs molecular chance of self-assembly and enhance its efficacy towards target binding (ergosterol), thus diminishing its toxicity [18].

### 3.3. Powder X-ray diffraction analysis

**Figure 4.**
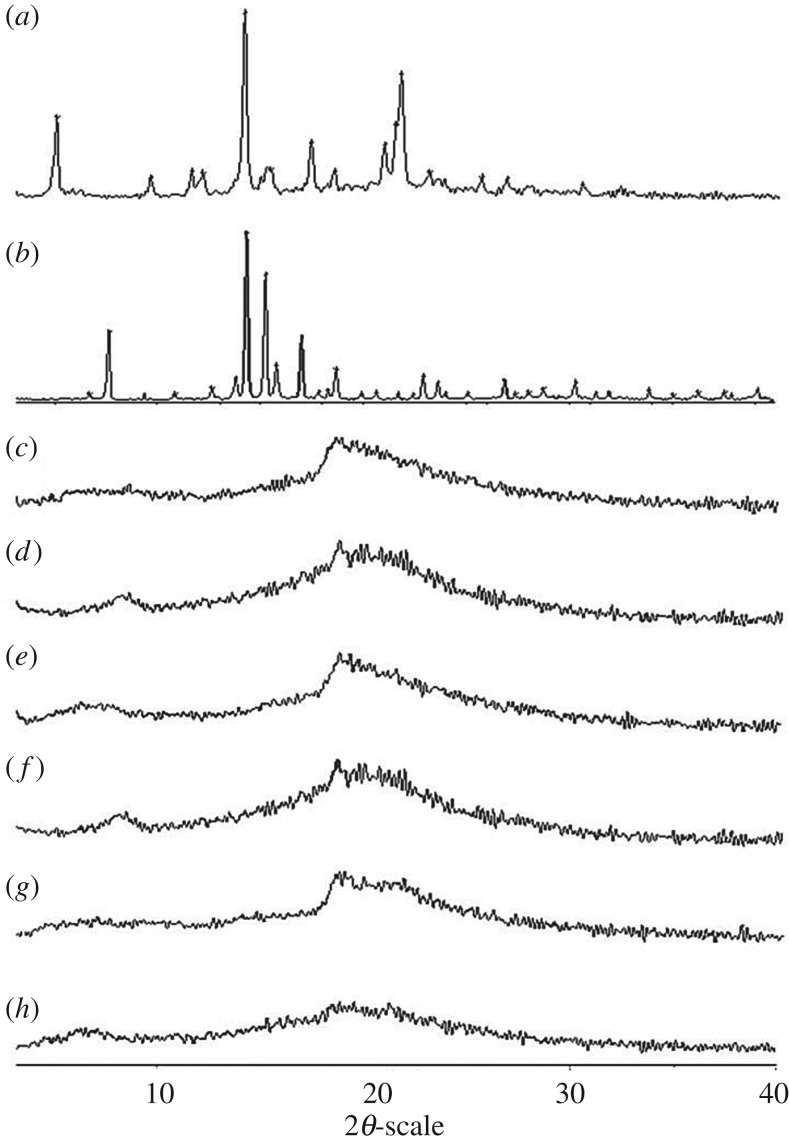
PXRD spectra of (*a*) AmB, (*b*) Dex, (*c*) Nap-FFDKY, (*d*) Npx-FFDKY, (*e*) Fmoc-FFDKY, (*f*) Nap-AmB, (*g*) Npx-AmB and (*h*) Dex-AmB compounds powder.

The PXRD analysis confirmed the crystalline nature of the AmB, Dex and the lyophilized powder of hydrogels (figure 4*a*,*b*). The PXRD of Nap-FFDKY, Npx-FFDKY and Fmoc-FFDKY lyophilized powder of hydrogels shows an amorphous halo (figure 4*c*,*d*,*e*). The PXRD of AmB exhibits a sharp peak at 2*θ* scattered angles of 14.06°, 17.29° and 21.65°. The PXRD of Dex exhibits a sharp peak at 2*θ* scattered angles of 7.59°, 14.28°, 15.21°, 18.63° and 22.88°. However, the characteristic peaks for AmB or Dex are absent in the PXRD spectra of Nap-AmB, Npx-AmB and Dex-AmB formulation hydrogels (figure 4*f*,*g*,*h*). Therefore, the results demonstrate that the crystalline structures of the AmB and Dex are changed after the formation of hydrogels. In previous reports, AmB had a natural ability to assemble spontaneously into higher order molecular forms that result in its toxic property [19]. The immobilization of AmB on a peptide substrate will minimize its chance of self-assembly. The amorphous hydrogels would enhance AmB efficacy towards binding of fungal ergosterol, diminish drug toxicity and minimize the chance of fungal resistance to AmB [20].

### 3.4. Circular dichroism spectra

**Figure 5.**
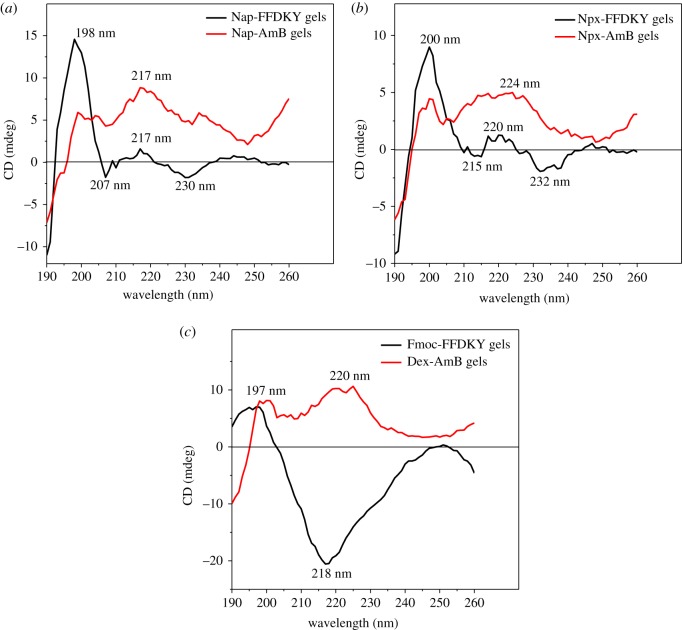
CD spectra of (*a*) Nap-FFDKY and Nap-AmB hydrogels; (*b*) Npx-FFDKY and Npx-AmB hydrogels; (*c*) Fmoc-FFDKY and Dex-AmB hydrogels (0.1–1 mg ml^−1^ in water).

The CD spectra of the solution of blank carriers and the corresponding drug deliveries further help elucidate the molecular arrangement in the gel phase. The CD spectrum of Nap-FFDKY hydrogels exhibits a positive peak near 198 nm, a broad positive band near 217 nm, a negative peak near 207 nm and a negative peak near 230 nm, indicating the existence of turn features. The positive peak of Nap-AmB hydrogels at 217 nm agrees with the Nap-FFDKY hydrogels in a turn secondary structure (figure 5*a*). For Npx-FFDKY hydrogels, the spectrum exhibits a positive peak near 200 nm, a broad positive band near 220 nm, a negative peak near 215 nm and a negative broad peak near 232 nm, indicating the existence of turn features. The positive peaks of Npx-AmB hydrogels at 200 nm and 232 nm are similar to those of the Npx-FFDKY hydrogels in a turn feature structure (figure 5*b*). Fmoc-FFDKY hydrogels exhibit a positive peak near 197 nm and a broad negative peak near 218 nm, indicating the existence of β-like features. Dex-AmB hydrogels exhibit different CD spectra. There are positive peaks near 197 nm and a broad positive peak near 220 nm, indicating the existence of turn features (figure 5*c*).

### 3.5. Drug release

**Figure 6.**
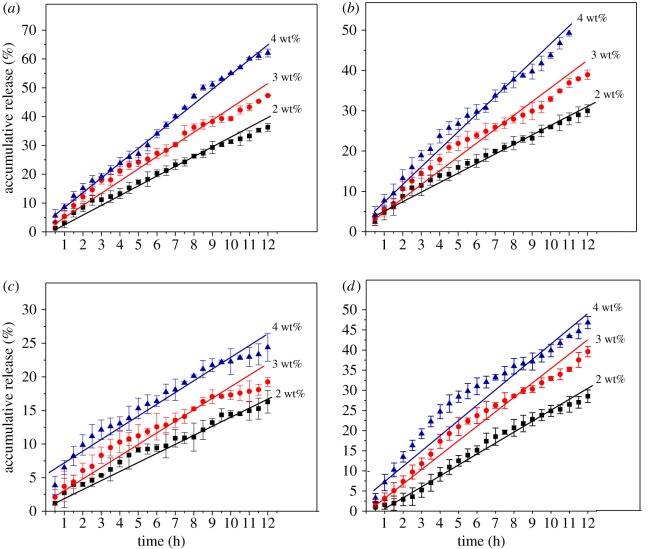
Accumulative release profile of AmB from (*a*) Nap-AmB, (*b*) Npx-AmB and (*c*) Dex-AmB hydrogels and Dex from (*d*) Dex-AmB hydrogels in PBS solutions at 37°C (pH = 7.4, *n* = 3).

The *in vitro* drug releases of drugs from the Nap-AmB, Npx-AmB and Dex-AmB hydrogels (2.0, 3.0 and 4.0 wt%) were investigated in the PBS solutions (pH 7.4) at a physiological temperature of 37°C. The cumulative release curves of AmB and Dex from the drug-loaded hydrogels are shown in figure 6. The release behaviour exhibits a sustained release mechanism within 12 h at a physiological temperature without burst releases. After 12 h, the final drug release percentages of AmB are 36.3%, 47.3% and 62.1% for 2.0, 3.0 and 4.0 wt% of Nap-AmB hydrogels, respectively (figure 6*a*). AmB is released from 2.0 wt%, 3.0 wt% and 4.0 wt% Nap-AmB hydrogels at a constant rate of about 0.3188, 0.6114 and 1.015 mg ml^−1^ per hour throughout the entire measurement period, respectively. Similarly, the final drug release percentages of AmB are 30.0%, 38.9% and 51.3% for 2.0, 3.0 and 4.0 wt% of Npx-AmB hydrogels, respectively (figure 6*b*). AmB is released from 2.0 wt%, 3.0 wt%, and 4.0 wt% Npx-AmB hydrogels at a constant rate of about 0.2406, 0.4837 and 0.8332 mg ml^−1^ per hour throughout the entire measurement period, respectively. Probably owing to the compactness of nanofibre networks, the final drug release percentages of AmB are 16.3%, 19.2% and 24.4% for 2.0, 3.0 and 4.0 wt% of Dex-AmB hydrogels, respectively (figure 6*c*). For Dex-AmB hydrogels, AmB is released from 2.0 wt%, 3.0 wt% and 4.0 wt% hydrogels at a constant rate of about 0.1123, 0.1994 and 0.3517 mg ml^−1^ per hour throughout the entire measurement period, respectively. As for Dex, the final drug release percentages are 28.5%, 39.6% and 46.8% for 2.0, 3.0 and 4.0 wt% of Dex-AmB hydrogels, respectively (figure 6*d*). Dex is released from 2.0 wt%, 3.0 wt%, and 4.0 wt% Dex-AmB hydrogels at a constant rate of about 0.08359, 0.1743 and 0.2867 mg ml^−1^ per hour throughout the entire measurement period, respectively.

According to the empirical equation, the exponential factor (*n*) has been evaluated to determine the mechanism by fitting the experimental data. For AmB released from hydrogels, the values of *n* are 0.6874, 0.5722 and 0.5481, for 2.0 wt%, 3.0 wt% and 4.0 wt% of Nap-AmB hydrogels, respectively. The values of *n* are 0.7428, 0.7784 and 0.7771 for 2.0 wt%, 3.0 wt%, and 4.0 wt% of Npx-AmB hydrogels, respectively. The values of *n* are 0.8265, 0.6474 and 0.7971, for 2.0 wt%, 3.0 wt% and 4.0 wt% of Dex-AmB hydrogels, respectively. The fitting values of *n* are between 0.5 and 1, indicating that the release mechanism of AmB is the non-Fickian diffusion. The results indicate the anomalous nature of drug release, to which both diffusion and relaxation processes contribute [21]. It may be due to the breakage of coordinate amide bonds between AmB drug and the peptide chains. For Dex released from Dex-AmB hydrogels, the values of *n* are 0.4404, 0.3576 and 0.2587 for 2.0 wt%, 3.0 wt% and 4.0 wt% of Dex-AmB hydrogels, respectively. The fitting values of *n* are lower than 0.5, indicating that the drug release follows the Fickian diffusion. It occurs by the usual molecular diffusion of the drug owing to a chemical potential gradient [22]. For Nap-AmB hydrogels, the rate constant values *k* of AmB released from 2.0 wt%, 3.0 wt% and 4.0 wt% hydrogels are approximately 0.2503 (*r* = 0.9165), 0.4662 (*r* = 0.9174) and 0.6379 (*r* = 0.9083), respectively. Similarly, the rate constant values *k* are approximately 0.2846 (*r* = 0.9263), 0.3358 (*r* = 0.9152) and 0.4387 (*r* = 0.9081) for 2.0 wt%, 3.0 wt% and 4.0 wt% Npx-AmB hydrogels, respectively. For Dex-AmB hydrogels, the rate constant values *k* of AmB released from 2.0 wt%, 3.0 wt% and 4.0 wt% hydrogels are about 0.2283 (*r* = 0.9041), 0.3669 (*r* = 0.9255) and 0.6436 (*r* = 0.9143), respectively. The rate constant values *k* of the Dex released from 2.0 wt%, 3.0 wt% and 4.0 wt% Dex-AmB hydrogels are approximately 0.1211 (*r* = 0.9104), 0.3039 (*r* = 0.9037) and 0.6024 (*r* = 0.9068), respectively. For all hydrogels, the rate constant value *k* approximately increases with increasing the content of hydrogels [23]. These results suggest that the Nap-AmB, Npx-AmB and Dex-AmB hydrogels are suitable as drug-controlled release vehicles for hydrophilic drugs AmB and Dex.

### 3.6. Antifungal effects

**Figure 7.**
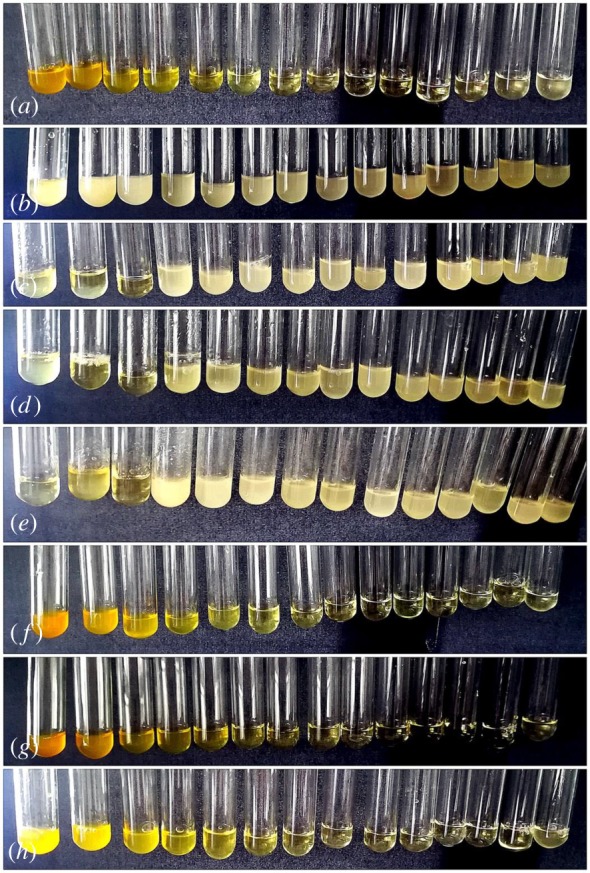
MIC test of AmB (2.078 mg ml^−1^ to 0.0002536 mg ml^−1^, *a*), Dex (5.190 mg ml^−1^ to 0.0006335 mg ml^−1^, *b*), Nap-FFDKY hydrogels (5.078 mg ml^−1^ to 0.0006198 mg ml^−1^, *c*), Npx-FFDKY hydrogels (5.048 mg ml^−1^ to 0.0006161 mg ml^−1^, *d*), Fmoc-FFDKY hydrogels (5.052 mg ml^−1^ to 0.0006168 mg ml^−1^, *e*), Nap-AmB hydrogels (5.475 mg ml^−1^ to 0.0006683 mg ml^−1^, *f*), Npx-AmB hydrogels (5.595 mg ml^−1^ to 0.0006830 mg ml^−1^, *g*) and Dex-AmB hydrogels (5.14 mg ml^−1^ to 0.0006274 mg ml^−1^, *h*) were determined by a serial 2-fold dilution of test samples.

The antifungal effects of AmB, Dex, carrier hydrogels and AmB-conjugated hydrogels were tested against *C. albicans* and evaluated by the value of MIC and MBC (figure 7). Results indicate that AmB exhibits the antifungal activity with an MIC value of 0.00406 mg ml^−1^ and an MBC value of 0.130 mg ml^−1^, which are consistent with the previous literature reports [24]. The Dex, Nap-FFDKY, Npx-FFDKY and Fmoc-FFDKY hydrogels are less antifungal effective against *C. albicans*. For blank carrier, the first three test suspensions contain fungal strain and Nap-FFDKY (5.08, 2.54 and 1.27 mg ml^−1^), Npx-FFDKY (5.05, 2.52 and 1.26 mg ml^−1^) and Fmoc-FFDKY (5.05, 2.53 and 1.26 mg ml^−1^) hydrogels with relatively high concentrations form semisolid gels in the tube. These hydrogels inhibit fungal growth which may be due to massive compact network structures or Asp-Lys (DK) antibacterial dipeptide fragments. Nap-AmB, Npx-AmB and Dex-AmB hydrogels significantly exhibit the antifungal activity with MIC values of 0.0107, 0.0437 and 0.0401 mg ml^−1^ respectively, and MBC values of 0.171, 0.349 and 0.643 mg ml^−1^ respectively. These findings illustrate an innovative strategy to significantly inhibit fungal strains at low amounts of AmB.

### 3.7. Time-kill curves

**Figure 8.**
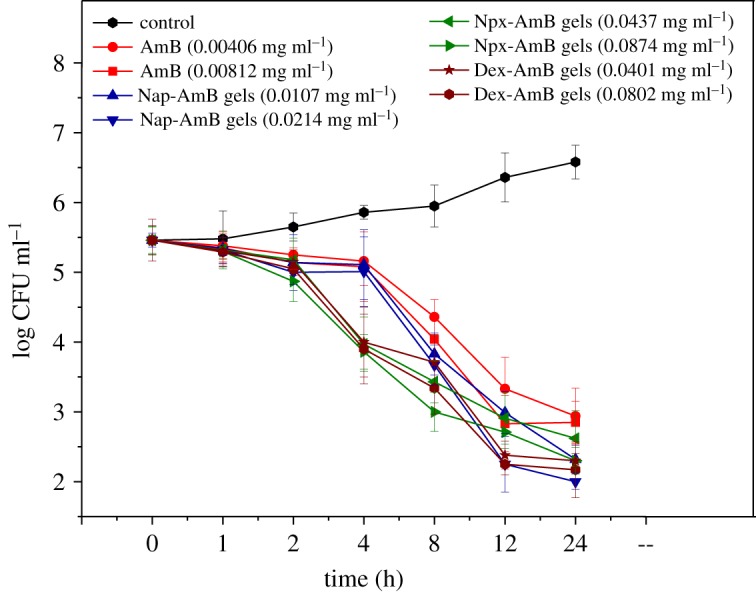
The time-kill curves of AmB, Nap-AmB hydrogels, Npx-AmB hydrogels and Dex-AmB hydrogels (*n* = 3).

Furthermore, we obtained detailed information about antifungal efficacy as a function of both time and concentration by time-killing tests (figure 8) to assess the pharmacodynamics of each antifungal agent. The concentration-dependent actions of AmB and AmB-conjugated hydrogels are confirmed by time-kill curves. Moreover, the curves also reveal the time-dependent action between them. The time-kill curves show the AmB and Nap-AmB, Npx-AmB and Dex-AmB hydrogels exhibit antifungal activity for 2 h. The Npx-AmB and Dex-AmB hydrogels exert fungistatic effect more quickly than AmB and Nap-AmB hydrogels. Discernible improvement in the extent of antifungal activity and the slope function of the time-kill curve to each sample is noted as the amount of drug in solution increased. Marked concentration-dependent antifungal activity was also observed, what is more, the rate and extent of antifungal activity varied over time and the time to achieve an antifungal endpoint was shortened as the dose increased. In addition, the AmB-conjugated hydrogels showed a striking improvement in the extent of activity and trend towards a shorter time to the antifungal endpoint versus AmB agents [25].

## 4. Conclusion

The present work is focused on the design and development of AmB conjugated to biocompatible and biodegradable polypeptide hydrogels to improve antifungal activity. Amphotericin-conjugated hydrogels are characterized for physicochemical properties and evaluated for *in vitro* antifungal activity. The results of the present study indicated that the AmB-conjugated hydrogels are suitable carriers for poorly water soluble drugs and for the enhancement of therapeutic efficacy of antifungal drugs.

## Supplementary Material

Chemical structures, MS spectra, 1H-NMR spectra, HPLC spectra and the antifungal effect of compounds or drugs.

## Data accessibility

Complementary information supporting this article has been uploaded as part of the electronic supplementary material.
