# Supplementary material for: Amphotericin B-conjugated polypeptide hydrogels as a novel innovative strategy for fungal infections
Source: R Soc Open Sci. 2018 Mar 14;5(3):171814. doi: 10.1098/rsos.171814 (PMC5882710; doi:10.1098/rsos.171814)
Supplement: Chemical structures, MS spectra, 1H-NMR spectra, HPLC spectra and the antifungal effect of compounds or drugs. [file rsos171814supp1.docx]

**Supporting Information**

**Fig. S1** Synthetic route of Nap-FFDKY compound.

**Fig. S2** MS spectrum of Nap-FFDKY compound.

**Fig. S3** ^1^H-NMR spectrum of Nap-FFDKY compound.

**Fig. S4** Synthetic route of Npx-FFDKY compound.

**Fig. S5** MS spectrum of Npx-FFDKY compound.

**Fig. S6** ^1^H-NMR spectrum of Npx-FFDKY compound .

**Fig. S7** Synthetic route of Fmoc-FFDKY compound.

**Fig. S8** MS spectrum of Fmoc-FFDKY compound.

**Fig. S9** ^1^H-NMR spectrum of Fmoc-FFDKY compound.

**Fig. S10** Chemical structure of Nap-AmB compound.


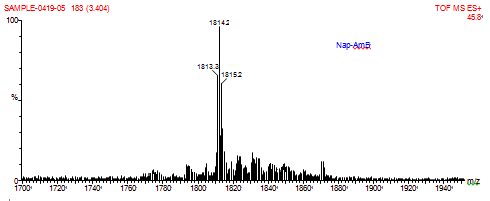


**Fig. S11** MS spectrum of Nap-AmB compound.

**Fig. S12** ^1^H-NMR spectrum of Nap-AmB compound.

**Fig. S13** Chemical structure of Npx-AmB compound.

**
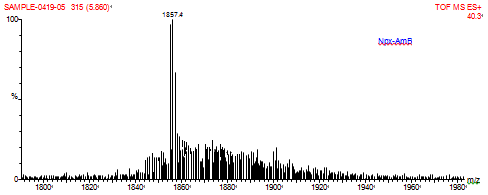
**

**Fig. S14** MS spectrum of Npx-AmB compound.

**Fig. S15** ^1^H-NMR spectrum of Npx-AmB compound.

**Fig. S16** Synthetic route of Dex-AmB compound.


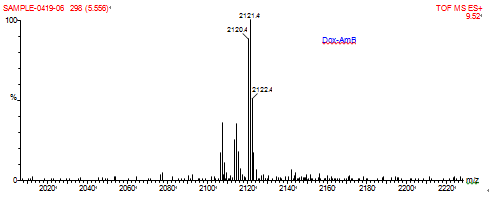


**Fig. S17** MS spectrum of Dex-AmB compound.

**Fig. S18** ^1^H-NMR spectrum of Dex-AmB compound.

**Fig. S19** The HPLC spectrum of AmB drug.

**Fig. S20** The HPLC spectrure of Dex drug.


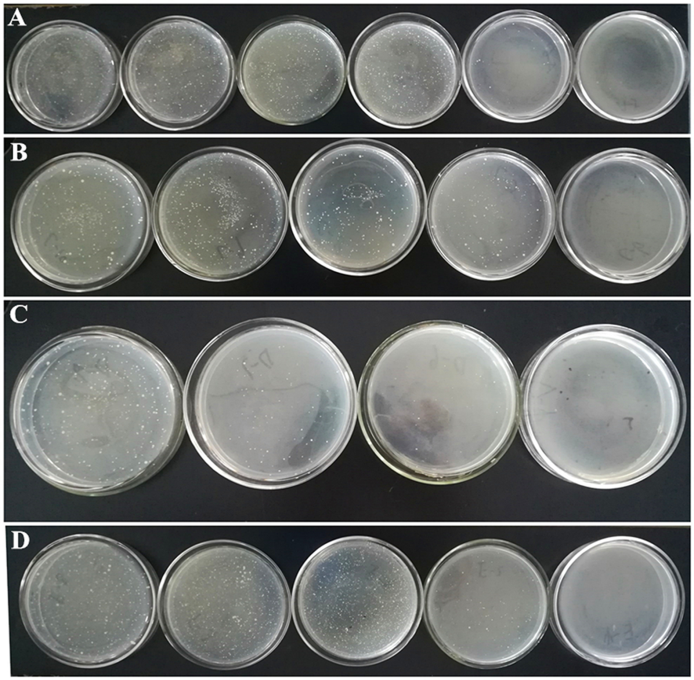


**Fig.S21** The antifungal effect of AmB and AmB-conjugated hydrogels were tested against Candida albicans. (A) The concentration of AmB is 0.00406 mg/mL (MIC), 0.00812 mg/mL, 0.0162 mg/mL, 0.0325 mg/mL, 0.0649 mg/mL, 0.130 mg/mL (MBC); (B) The concentration of Nap-AmB hydrogels is 0.0107 mg/mL (MIC), 0.0214 mg/mL, 0.0428 mg/ mL, 0.0855 mg/mL, 0.171 mg/mL (MBC); (C) The concentration of Npx-AmB hydrogels is 0.0437 mg/mL (MIC), 0.0874 mg/mL, 0.175 mg/mL, 0.349 mg/mL (MBC); (D) The concentration of Dex-AmB hydrogels is 0.0401 mg/mL (MIC), 0.0803 mg/mL, 0.161 mg/Ml, 0.321 mg/mL, 0.643 mg/mL (MBC).
